# Hyperphosphatemia during spontaneous tumor lysis syndrome culminate in severe hypophosphatemia at the time of blast crisis of Ph^neg^ CML to acute myelomoncytic leukemia

**DOI:** 10.1186/2162-3619-1-24

**Published:** 2012-08-29

**Authors:** Ophira Salomon, Eli J Holtzman, Pazit Beckerman, Camila Avivi, Luba Trakhtenbrot, Abraham Kneller, Tali Tohami, Yeroham Kleinbaum, Sara Apter, Ninette Amariglio, Ehud Grossman, Ginette Schiby

**Affiliations:** 1 The Amalia Biron Research Institute of Thrombosis and Hemostasis, Sheba Medical Center and Sackler Faculty of Medicine, Tel Aviv University, Tel Aviv, Israel; 2 Institute of Nephrology and Hypertension, Sheba Medical Center and Sackler Faculty of Medicine, Tel Aviv University, Tel Aviv, Israel; 3 Department of Pathology, Sheba Medical Center and Sackler Faculty of Medicine, Tel Aviv University, Tel Aviv, Israel; 4 Hematology Laboratory, Sheba Medical Center and Sackler Faculty of Medicine, Tel Aviv University, Tel Aviv, Israel; 5 Institute of Hematology, Sheba Medical Center and Sackler Faculty of Medicine, Tel Aviv University, Tel Aviv, Israel; 6 Department of Diagnostic Imaging, Sheba Medical Center and Sackler Faculty of Medicine, Tel Aviv University, Tel Aviv, Israel; 7 Department of Internal Medicine D, Sheba Medical Center and Sackler Faculty of Medicine Tel Aviv University, Tel Aviv, Israel

**Keywords:** Acute leukemia, Tumor lysis syndrome, Apoptosis, Hypophosphatemia, Hyperphosphatemia

## Abstract

Extreme swing of phosphor from severe hyperphosphatemia to severe hypophosphatemia in a patient with blast crisis of myeloid origin was the result of imbalance between massive apoptosis of leukemic cells in the context of spontaneous tumor lysis syndrome and massive production of leukemic cells with only 1% of blast in peripheral blood. The mutated p53 protein suggested acting as oncogene in the presented case and possibly affecting phosphor status.

## Background

Severe symptomatic hypophsopahatemia was reported in a small number of case reports in patients with acute myelomonocytic leukemia [1] with extreme white blood count (200-380/μL) because of high phosphate demands of dividing leukemic cells. It was also reported during hematopoietic reconstitution after allogeneic peripheral blood stem cell transplantation [2] due to excessive cellular phosphate uptake for hematopoietic reconstitution and in tumor genesis [1].

Leukemic blast cells of myeloid origin fail to show inhibition of glycolysis by oxygen [3] and causes hypophosphatemia by redistribution of phosphorus to the cells, to provide phosphate for glycolytic intermediates.

Severe hypophosphatemia may adversely affect major organ systems including severe generalized muscle weakness as observed in our patient. Reduction in the erythrocyte content of 2,3-diphosphoglycerate may impair release of oxygen resulting in hypoxia [4]. On the other hand hyperleukocytosis can cause pseudohyposphatemia, pseudohypokalemia [5] and pseudohypoxaemia. Unawareness to this phenomenon can lead to incorrect and harmful treatment.

## Case presentation

An 80-year-old white Ashkenazi Jewish woman presented at hematology out-patient clinic with persistent leukocytosis for the last 10 months. A peripheral blood count revealed white-cell count (WBC) of 36,000/μL with 58% neutrophils, 14% lymphocytes, 4% monocytes, 1% blasts, 2% metamyelocytes, 6% myelocytes and 10% segments. The hemoglobin was 12gr/dL, platelet counts 476,000/μL and blood chemistry was unremarkable. Her physical examination was normal as well as whole body computerized tomography scanning (CT). Bone marrow (BM) aspiration at that time was hyperplastic, enriched with white cells with normal maturation and no blasts. Megakaryocytes had dysplastic features with single nucleous without lobulations. The BM findings were compatible with a myeloproliferative disorder with some dysplastic changes. Cytogenetic analysis revealed 47XX in 75% of the cells with trisomy 8 (+8) detected also by fluorescent in situ hybridization. Janus Kinase 2 gene V617F mutation and Bcr/Abl fusion gene were not detected.

At that point, the patient's diagnosis was Philadelphia negative (Ph ^neg^) chronic myelogenous leukemia (CML) with+8. Of note that Bcr/Abl negative CML is clinically distinct from Bcr/Abl positive CML, myelodysplastic syndrome and chronic myelomonocytic leukemia [6] and usually, is associated with poor prognosis [7,8]. One could argue whether atypical CML, Bcr/Abl negative according to WHO classification could represent chronic neutrophilic leukemia as in the presented case.

**Figure 1:**
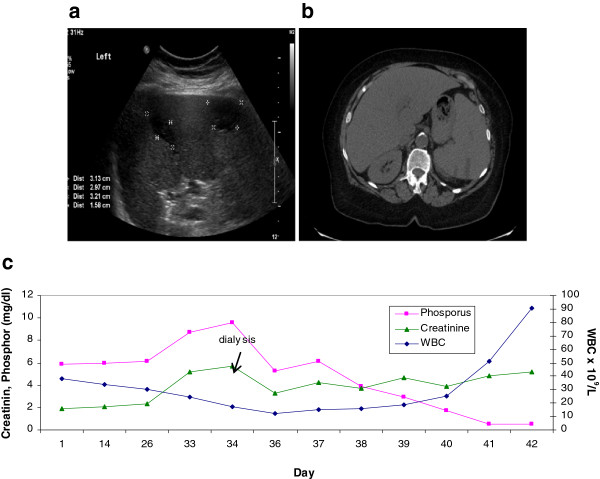
**a: Grey scale image of ultrasound of the spleen demonstrate small peripheral hypo echoic area compatible with infarcts (short arrow).b**: Non enhanced CT image of the spleen demonstrate hypodense peripheral areas compatible with splenic infarcts (arrow). **c**: Level of phosphor in peripheral blood in correlation to white cell counts. The extreme shift from hyperhsophatemia to severe hypophsphatemia is demonstrated.

A year and a half after diagnosis of Ph^neg^ CML was established, the patient complained of persistent discomfort and pain in the upper left abdomen lasting three days without fever. Laboratory work-up showed creatinine of 1.9 mg/dL, uric acid 13.5 mg/dL, phosphor 5.9 mg/dL, potassium 4.8 meq/L and LDH 403 IU/L. White blood cell were 38,000/ μL with 76% neutrophils, 7% lymphocytes, 17% monocytes , 1% blasts, 4% metamyelocytes, 10% myelocytes and 9% segments. The hemoglobin was 10 gr/dL and platelet count was 243 k/ μL. Abdominal ultrasound (US) Doppler revealed splenomegaly with several peripheral hypoechogenic areas compatible with spleen infarcts (Figure 1a). Also, a small calculus in lower calices of left kidney was noted. Abdominal CT scan demonstrated few peripheral hypodense areas compatible with spleen infarcts (Figure 1b). No enlargement of retroperitoneal lymph nodes or bulky mass was observed.

A month earlier, the patient went through colonoscopy and a villous adenoma polyp was found. Urianalysis of sediment revealed amorphous urates and 30 erythrocytes/ high-power field (HPF).

A rise in creatinine at the time of presentation from 0.65 to 1.9 mg/dL, uric acid up to 13.5 mg/dL, phosphate up to 5.9 with calcium 9.6 mg/dL, LDH 403 IU/L and alkaline phosphatase 57 IU/L support a diagnosis of acute urate nephropathy. The latter is the outcome of precipitation of uric acid in distal tubules and collecting ducts because of increased filtered load of urate due to excessive production of purines as occurs in tumor lysis syndrome (TLS) [9]. TLS can occur spontaneously [10], but it is commonly seen following the initiation of anti-cancer treatment [9]. It occurs in both hematological malignancies and in solid tumors, which harbor high proliferative rates and tumor burden.

The patient's creatinine continued to rise, and the phosphor reached a value of 9.6 mg/dL. At that point the patient was started on hemodialysis and received 5 treatments, before further deterioration occurred. The patient was also treated with aluminum hydroxide (950 mg tid).

She had hyperuricemia and severe hyperphosphatemia when neither chemotherapy nor biological agents or steroids were administered. Furthermore, there was no evidence of a bulky disease. Patient's counts remained stable. Although the patient's ultrasound study revealed a kidney stone, there was no evidence of any ureteral obstruction to explain the onset of renal failure**.** The use of oral sodium phosphate solution preparations for bowel preparation before colonoscopy could cause acute renal failure because of high phosphorus content that potentially can cause chronic kidney damage [11] but she was not treated with phosphate based preparations and the hyperphosphatemia developed a month after the procedure. One should also consider renal infarcts in the differential diagnosis of the patient's renal failure taking also into account the assumption of spleen infarcts by US and CT scan. However kidney infarcts were ruled out using dynamic and static kidney scintigraphy with Tc-99 M-DTPA. Doppler US of renal arteries ruled out other renal arterial pathology.

**Figure 2:**
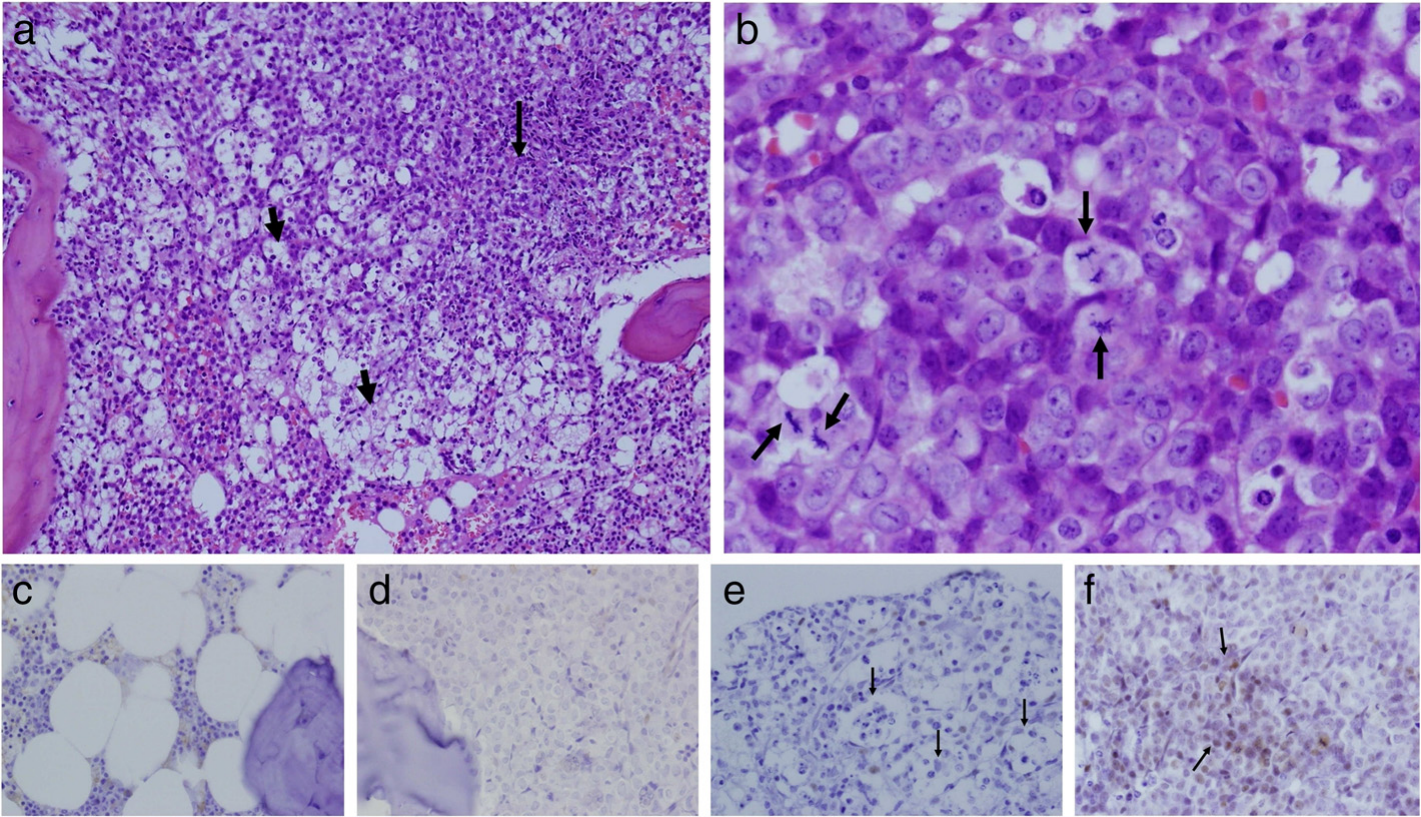
**a. The white areas represent extreme apoptosis of the leukemic cells, up to disintegration of the tissue (short arrows).** Right to these areas (arrow), viable and proliferating leukemic cells are present (H&E, 20x10). **b**. Higher magnification of viable and proliferating leukemic cells. Mitotic figures are indicated by arrows (H&E, 60x10). **c**. Normal bone marrow as negative control (p53 phosphor-specific antibody immunostain, 60x10). **d**. Bone marrow with AML as negative control (p53 phosphor-specific antibody immunostain, 60x10). **e**. The apoptotic and disintegrated areas, indicated by arrows, are negative (p53 phosphor-specific antibody immunostain, 60x10). **f**. The viable and proliferating areas are positive. See arrows (p53 phosphor-specific antibody immunostain, 60x10).

At this point, monocytosis in peripheral blood counts became significant and anemia was evident. Histopathology of the BM showed hypercellularity with sheets of immature-looking cells comprising at least 90% of the BM spaces. The red cell line and the megakaryocytes were inconspicuous. Extensive apoptosis of the cells with phagocytosis and a “starry sky” appearance, reminding Burkitt’s-like lymphoma was a prominent feature (Figure 2, a+b). On immunostains, the immature-looking cells were positive for myeloperoxidase and negative for CD20, CD3, CD10, BCL6, BCL1, Tdt and CD34. The CD68 indicated the presence of numerous histiocytes, but the immature-looking cells were negative. Immunophenotyping of BM disclosed 21% blasts in the blast gate with features of myeloblast/monoblast (HLADR, CD14, CD33, and CD64). None of the cells were CD34 positive. According to the morphological and phenotypical features the diagnosis of myelomonoblastic leukemia was made. The patient was treated with hydroxyurea for 5 days only until further deterioration occurred.

Constitutional activation of the MYC proto-oncogene resulting from a t(8;14) has been demonstrated in approximately 80% of patients with Burkitt lymphoma, but only in two cases with acute myeloid leukemia (AML) [12]. Search for t(8;14)/cMyc was negative albeit the histology appearance of "starry sky". Interestingly +8 which is among the commonest genetic aberrations (70%) seen in AML [13] declined from 90% to 24% suggesting the emergence of a different clone at the time of transformation into acute leukemia.

The acute myelomonocytic leukemia that developed in our patient might explain the relative hypokalemia in spite of tumor lysis and renal failure. Hypokalemia is known to develop in patients with acute monoblastic leukemia and significantly correlates with hyperleukocytosis , increased blood and urine lysozyme. The lack of hyperkalemia initially raised questions regarding the diagnosis of TLS and prompted the aforementioned investigation for other etiologies of renal failure.

In a three week period the patient developed weakness and fatigue which led to diagnosis of severe hypophosphatemia <0.5 mg% (Figure 1c) while potassium remained in normal level despite the presence of progressive renal failure. Other test results revealed: serum calcium between 10.6-11.2 mg%, venous pH 7.38, venous lactate levels mostly within normal limits except for one elevated measurement of 30 mg% (N 6-18 mg%). Aluminum hydroxide was stopped and the patient was treated with IV KPO4 with no significant improvement.

Several potential causes that could contribute to hypophosphatemia in our patient were ruled out, such as nutritional causes, antacids ingestion, sepsis or diabetic ketoacidosis. Urine output was low, and parathyroid hormone and 1,25(OH)2 dihydroxyvitamin D levels were within normal limits, thereby excluding overt hyperparathyroidism and oncogenic hypophosphatemic osteomalacia even though, urine phosphor levels were not measured.

In addition the patient was also never treated with chemotherapeutic agents beside hydroxyurea or thyrosine kinase inhibitor such as imatinib, known to cause mild hypophosphatemia (around 2 mg%), possibly through inhibition of bone turnover, which in turn, triggered a secondary hyperparathyroidism in an attempt to maintain calcium homeostasis [14]. Another suggested mechanism is depletion of intracellular phosphate in renal tubular cells, potentially interfering with reabsorption of urinary phosphate [15].

In our patient the shift of phosphor from severe hyperphosphatemia of 9.6 mg/dL to <0.5 mg/dL phosphor six days later even when WBC counts solely tripled in number (Figure 1c) and analysis of blood phosphor was done immediately in a patient with end stage renal failure was the warning sign of the arrival of fulminant and aggressive leukemia without more than 1% of blasts of peripheral WBC at that time of demise.

The presence of massive apoptosis was noted aside to tumor genesis in the patient's BM (Figure 2) and it is conceivable that the phosphor that is released during apoptosis is reuptaken by cells for tumor genesis, explaining the extreme change in phosphor level up to severe hypophosphatemia in our patient. Therefore, we determined to assess whether the missing phosphor could be seen in patient's BM specimen. Although there is no technique quantifying free phosphor but in serum, we tried to asses intracellular phosphor content via immunohistochemical stain. We chose p53 phosphor-specific antibody (clone EP42Y, Burlingame, California) due to the fact that in the presence of wild P53 protein, tumor cells will usually go into apoptosis in contrast to mutated P53 protein where it can act as oncogene thus promoting tumor genesis [16]. The unmutated p53 protein, when phosphorylated on serine-46 site (pS46), becomes a stabilized and activated proapoptotic protein, induced by DNA-damage. But the p53 phosphor-specific antibody that we used stained negative in apoptotic cells and positive in part of the viable proliferating blasts suggesting that the P53 was mutated (Figure 2).

## Conclusions

The presented case demonstrates the extreme shift from severe hyperphsosphatemia to severe hypophosphatemia during blast crisis of a patient with Ph^neg^ CML. Hyperphosphatemia was due to massive apoptosis of leukemic cells in the context of TLS and then inverted to severe hypophosphatemia despite end stage renal failure when tumor genesis prevailed and resulted in the patient's death with peripheral WBC of 132,000 /uL, of those only 1% were blasts. Since neither the occurrence nor the severity of this hyper-hypophosphatemic shift can be predicted from the patient’s baseline disease, and therefore cannot be treated ahead of time, it is very important to be aware of this possible complication, carefully follow-up serum phosphor and provide immediate treatment when even mild changed occur.

## Consent

Institutional Helsinki approval, no. 9829-12-SMC.

## Competing interests

The authors declare that they have no competing interest.

## Authors’ contributions

OS – designed and wrote the manuscript. EH – treated patients, designed and wrote the manuscript. PB – was involved in writing the manuscript. CA – performed the immunohistochemical stains of all pathology sections. LT – performed the FISH analysis. AK – treated the patients and analyzed the results. TT – performed the PCR work. YK – performed the ultrasound study and analyzed the results. SA – performed the CT scan and analyzed the results. NA – involved in PCR and FISH tests and analysed the results. EG designed the study, participated in the sequence alignment and in writing the manuscript. RS – performed all pathology sections, analysed the data and was involved in writing the manuscript. All authors read and approved the final manuscript.
